# A commentary on the carbon footprint of milk formula: harms to planetary health and policy implications

**DOI:** 10.1186/s13006-019-0243-8

**Published:** 2019-11-27

**Authors:** Julie P. Smith

**Affiliations:** Research School of Population Health, Australian National University, Canberra, Australia

## Abstract

**Background:**

Infant formula requires mass production by the dairy industry, with plastic and other waste and degradation of land and waterways. Millions of babies, two thirds globally, now have milk formula, with breastfeeding in dramatic decline in Asia.

**Economic cost externalities and commercial incentives:**

Economic thinking clarifies that markets are not perfect - price incentives can lead to excessive and inefficient environmental damage. Market prices paid to produce or use a commodity may not reflect its true resource costs. The ongoing global transition in infant and young child feeding (IYCF) toward milk formula use makes urgent the investigation of its environmental costs, including greenhouse gas (GHG) implications. Socially vulnerable populations are also particularly exposed to climate change risks, but have the least voice and agency.

**The important role of public health advocacy:**

Few question the scale of the baby food industry, especially in major food exporting countries. Breastfeeding advocacy non-government organisations have led the investigations, and exposed the inequitable vulnerabilities. A ground-breaking study in 2016 showed emissions from just six Asia Pacific countries were equivalent to 6 billion miles of car travel. Each kilogram (kg) of milk formula generated 4 kg of (carbon dioxide (CO_2_) equivalent) greenhouse gas during production. Much of this was from unnecessary toddler formula. Recent research reveals that if looking at the full product lifecycle, including consumer use, GHG emissions per kg are actually three times higher than these pioneering estimates. Environment and health harms combined with economic evidence highlight the place for a strong public health response on this issue.

**Conclusion:**

Formula feeding is a maladaptive practice in the face of contemporary global environmental and population health challenges. Breastfeeding protection, support and promotion helps to safeguard planetary and human health by minimising environmental harm. It is a beneficial response to concerns about disease burdens and climate change. Breastfeeding populations are more resilient in emergencies. Effective and cost-effective policies and interventions exist for increasing breastfeeding and reducing unnecessary use of formula. Implementing such measures presents a rare opportunity to both reduce the greenhouse gas problem and improve human nutrition, health, and health equity.

## Background

Baby milk formula requires mass production by the dairy industry, with plastic and other waste and degradation of land and waterways. Millions of babies, two thirds globally, now have milk formula, with breastfeeding in dramatic decline in Asia. The scale of the damage to planetary health now revealed by studies of the resulting greenhouse gas emissions is staggering, and warrants urgent action to protect breastfeeding in all country settings.

In December 2015, governments meeting in Paris agreed on doing something about climate change [1]. Negotiations were high level, and the links between human health, climate change and the environment hardly rated a mention amidst the detailed accounting for lost profit and reduced emissions. No one mentioned the world’s mothers and babies, or the environmental consequences of how most of the infants and children are now fed. Nearly 5 years later little has changed.

Around two thirds of humans are inappropriately fed processed food in early life. Much commercial baby food is manufactured in countries which are hundreds, even thousands of miles away from where it is sold. Milk formula includes products called ‘infant formula’ as well as ‘follow-up’ formula and so called ‘growing up milks’ including toddler milk formula. Much comes from milking another species - a cow - grazed on watered pastures created from clearing forested land. If the resource costs of formula feeding were properly accounted for, the baby food industry would be scaled right back to producing milk formula as an essential remedy for rare circumstances, and mothers supported, even paid, to breastfeed. Economists call such unattributed resource costs ‘externalities’. At present, industry can ignore these costs, as they are instead passed on to society, or inflicted on the environment. The important but apparently invisible consequence is that disasters and emergencies are increasingly common, and when they happen, even more formula is shipped in, increasing environmental burdens further as breastfeeding is undermined by its indiscriminate use and by the ensuing creation of new markets for milk formula.

### The simple economics of externalities and market failure

Economic thinking clarifies that market failures may generate price incentives which result in excessive and inefficient environmental damage, because prices paid to produce or use a commodity may not reflect its true resource costs. Milk formula harms the environment not only through land clearing, water use, pollution and methane gas from dairy farming, but also through energy and water waste during manufacturing and distribution, and then due to packaging, food waste, and water and energy use to ensure hygiene. It increases maternal and child illness and chronic disease and thereby health treatment costs borne by families, insurers and taxpayers. As these ‘cost externalities’ fall substantially on parties other than the producer or purchaser, their decisions will tend to ignore such costs. Hence, production and use will be wasteful and excessive compared to what is socially optimal and efficient.

By distorting incentives, ‘externalities’ mean markets fail to give producers and consumers the right signals to conserve our common resources. In the case of mothers and babies, this means suboptimal nutrition resulting in higher health care costs for society, as well as for families. There is growing evidence of this [2–5]. Recent economic studies have highlighted the global economic costs of not breastfeeding, for countries and health systems, as well as for women and families [6, 7].

Likewise, when environmental consequences of the dairy industry are not fully accounted for in business decisions, costs are imposed on communities, increasingly on humanity as a whole, by greenhouse gas emissions, land and water degradation, and loss of biodiversity. Methane gas emanating from dairy herds is one of the most potent forms of greenhouse gas, and is a key element of the global greenhouse gas problem. Methane gas results in more damaging effects than other emissions. The greenhouse gas emissions for every kilogram of raw milk are substantial as it is made from dairy milk. Clearing the land for dairy farming creates its own damage to environmental diversity as well as in removing the Earth’s innate ‘carbon sinks’: its forests.

The contribution of modern food and animal agriculture systems to both greenhouse gas emissions and human health risks was apparent more than a decade ago [8, 9]. Meat and dairy corporations are now catching up with oil companies as the largest global emitters of greenhouse gases [10].

### The global transition in infant and young child feeding

A 2016 study published in *Public Health Nutrition* drew attention to the unprecedented global transition to milk formula for infant and young child feeding [11]. This has been particularly evident in the Asia Pacific region, and foreshadows significant future population health issues as well as adverse macroeconomic and health cost impacts due to detrimental effects on maternal and child health and development [12–15]. The boom in milk formula sales in these emerging middle-income countries is attributed to factors including lack of maternity protection for breastfeeding, unregulated company marketing of baby foods, and inadequate support for breastfeeding by health services and health professionals. Companies themselves are well aware of sales opportunities from delaying or weakening supportive breastfeeding policies, such as paid maternity leave or implementation of the Baby Friendly Hospital Initiative (BFHI). Baby food industry reports for individual countries reveal that companies promote baby food products such as ‘toddler formula’ to target market demand created by ‘busy lifestyles’ and ‘time pressed mothers’. Promotion also targets health professionals and facilities, to circumvent restraints on advertising to the public. Governments generally respond only belatedly to such emerging challenges to optimal breastfeeding practices.

A growing literature identifies the food system as a major contributor to climate change [16]. Along with escalating demand for meat and dairy products by more affluent populations particularly in Asia, the ongoing boom in sales of formula milk is forcing the global community to ask some hard questions about how to share the costs of climate change, across countries, within countries, and between the current and future generations. Decisions on climate change are politically difficult because the solutions may imply lower standards of living and health, which in practice are often inflicted on the poorest and weakest in society.

The answers to the problem of climate change are not simple, but at least in 2015 our leaders began asking some of the right questions. It is no longer, who profits versus who loses from the status quo, but rather, can humanity afford to continue with the status quo?

### Infant and young child feeding and the climate change debate

In the case of infant and young child feeding, those asking the right questions about links with climate change are yet to be heard by the global community. To question the scale and activities of the highly profitable baby food industry is still economic heresy, especially in major food exporting countries such as Australia or New Zealand [17].

Meanwhile the highly sustainable and efficient food system provided by lactating women for infants and young children through breastfeeding is invisible, unvalued and disrupted by food policies [18]. National and global leaders including in emerging economies such as China want to develop domestic dairy industries, but ignore the wider health and environmental costs of milk formula displacing breastfeeding. Maternal investments in breastfeeding such as through time, skill and energy in practicing and supporting it, are unmeasured, invisible and taken for granted, a mothers’ capacity to provide care for others is treated as limitless, because women’s time is seen as free [19].

Ironically, even as climate change contributes to extreme weather events, and greenhouse gas emissions from milk formula adds further to global climate change, mothers and their young children face heightened risks of insecurity and starvation due to changing weather patterns and related floods, droughts, fires, storms and other crises. The most vulnerable to the food insecurity, ill health and disease arising from greenhouse gas emissions, environmental damage, and climate change are those also exposed to formula and bottle feeding, often the poor living in exposed locations where land is cheaper.

High breastfeeding populations are more resilient in emergencies, lactating mothers provide the essential and only truly safe nourishment for human infants [20]. Supporting cross nursing and milk sharing when a child’s own mother is unavailable or unable to breastfeed, a practice well evidenced in human history, can provide life-saving potential during humanitarian emergencies and natural disasters [21, 22].

### NGO advocacy on infant feeding and climate change

A 2015 report, published by International Baby Food Action Network and the Breastfeeding Protection Network of India, with support from the Swedish and Norwegian development assistance agencies, initiated an important conversation about the milk formula industry’s contribution to greenhouse gas emissions and climate change [23]. The study provided alarming new data on how the present formula feeding epidemic ‘costs the earth’ in the form of greenhouse gas emissions, and dangerous climate change.

The report's analysis of public policy in the six study countries also revealed that governments, who should be initiating effective action to protect, promote and support breastfeeding, remained in deep paralysis on this issue. This problem has been highlighted in countries such as Australia, New Zealand, the United States, China and East Asia, where the policy conflicts with government’ goals for industry development or labour market expansion are not hard to find [24].

The 2015 study calculations showed that the GHG emission for a kilogram of milk formula (including standard infant formula, follow on formula, and toddler milk), was around 4 kg (CO_2_ equivalent). This excluded emissions after the manufacturing stage, such as arising due to packaging, transport, preparation and refrigeration of milk formula products. On this basis, milk formula sales totalling 720,450 tonnes in 2012 for six countries in our region (Australia, China, Malaysia, India, Philippines and South Korea), generated GHG emissions of around 2.89 million tonnes (CO_2_ equivalent). This was equivalent to driving 6888 million miles in the average US passenger car, sending around 1 million tons of waste to landfill, consuming 323 million gallons of gasoline or burning 3107 million pounds of coal.

The largest product contribution to these GHG emissions was toddler formula, which contributed 1.27 million tonnes of GHG (CO_2_ equivalent) in the six countries. Sales of standard infant formula and follow-on formula contributed just under a million tonnes each to GHG emissions.

Toddler milk is a product viewed by health authorities as both unnecessary and possibly harmful to child health [25, 26], yet has been underpinning rising sales of milk formula sales in both industrialised countries like Australia and emerging countries such as China [11, 12].

The greatest generation of GHG emissions from milk formula is in China, which has a large population of infants and young children, and dramatically declining breastfeeding rates [27]. In China, GHG emissions were 2.2 million tonnes (CO_2_ equivalent) in 2012 and were forecast in the IBFAN report to rise to 4.2 million tonnes by 2017. Australia, which is a major exporter of milk formula to China, generated around 32 thousand tonnes of GHG (CO_2_ equivalent) from its own use of these products.

This 2015 NGO study was described during the 2018 controversy on President Trump’s WHA intervention as a ‘ground-breaking’ report [28]. Long held concerns about the environmental impacts of the formula milk industry [29, 30] were translated into detailed data and analysis using contemporary scientific method. It was an important wake-up call.

### Climate change, infant feeding and WHO scientific experts

Just 4 years later, in 2019, the news on climate change is many times worse, but at least the World Health Organization (WHO) is paying attention to the issue of milk formula. At the current level of governments commitments to reduce greenhouse gas emissions, the world is set for a disastrous 3 °C of warming unless effective action is taken within 11 years [31, 32].

The 2015 IBFAN report estimated the GHG emissions from manufacturing 1 kg of infant or toddler milk to be around 4 kg of CO2 eq.; this was a partial estimate of the product’s lifecycle, and excluded carbon footprints at retail and household use stages [28]. In early 2019, a major study by World Health Organization and other researchers and funded by the Gates Foundation showed the IBFAN report to be highly conservative [33].

This 2019 study estimates the GHG of infant formula for the whole of the product lifecycle. Its shocking result is that a kilogram (kg) of infant formula adds between 11 and 14 kgs CO_2_ eq. of GHG to the planet by the time it is fed to babies and young children [33]. Sterilization, necessary as it is, adds significantly to post-factory environmental costs (which also include post-manufacture transportation, preparation and waste). Breastfeeding is far better for the environment, even if one allows for exclusively breastfeeding mothers eating more. The implication is that feeding an infant on milk formula for 6 months instead of exclusive breastfeeding requires 21 kg of formula, and adds over 200 kg of CO_2_ eq GHG emissions to the deteriorating future prospects for planetary and human health. For 2012, this would multiply at least threefold the carbon footprint estimated by the IBFAN research team [23]. Rather than 6 billion miles driving in a car, the 2012 emissions from the six Asia Pacific countries studied would have been estimated at in excess of 18 billion miles of car travel.

Most worryingly, by 2019, China alone accounts for emissions on this scale, due to the massive decline in breastfeeding and associated shift to milk formula in that country since 2012 [27, 34]. Less than a third of the 15 million babies born in China each year are exclusively breastfed. Recent sales data shows that, around 660,000 tonnes of powdered milk formula are now sold each year in China alone. At least half of this is so-called ‘growing up’ or ‘follow up’ milk formula, stated by WHO to be unnecessary and possibly harmful to the nutrition of young children [26]. The market prospects for these ‘premium’ (meaning high-priced) products are lauded by industry analysts. The products cater to demand created by ‘life pressures’ such as on mothers returning to work, and the ‘health’ marketing appeal of these industrialised milk powder products [35]. Such markets are in effect ‘created’ for industry by policies which fail to ‘internalise’ the cost externalities of baby milk formula products or to provide sufficient paid leave to enable breastfeeding.

### Public health and public responsibilities

Public health advocates in Australia and New Zealand [17, 36] have drawn attention to the responsibilities of regulators in Australasia to these trends, to no avail [24, 37]. The reality is that policies which fail to protect breastfeeding in a timely fashion from emerging market trends promote baby milk formula sales [38, 39].

Evidence on the wider, environmental harms of the global epidemic of formula feeding herald difficult decisions for governments in formula exporting and importing countries.

This is not about charging mothers as ‘guilty’ about infant and young child feeding practices. ‘Choice’ is the language of markets and marketing, but women’s decision-making on infant and young child feeding is far more complex. Individuals may in reality have far less influence on infant feeding than the public policies and health institution practices in the country they live in [12]. Wider public policy and institutional frameworks are now well understood to be important for individual women’s and families’ decisions and practices. Responsibility lies with policymakers, as much as with parents.

### Commercial determinants of infant and young child feeding

**Fig. 1:**
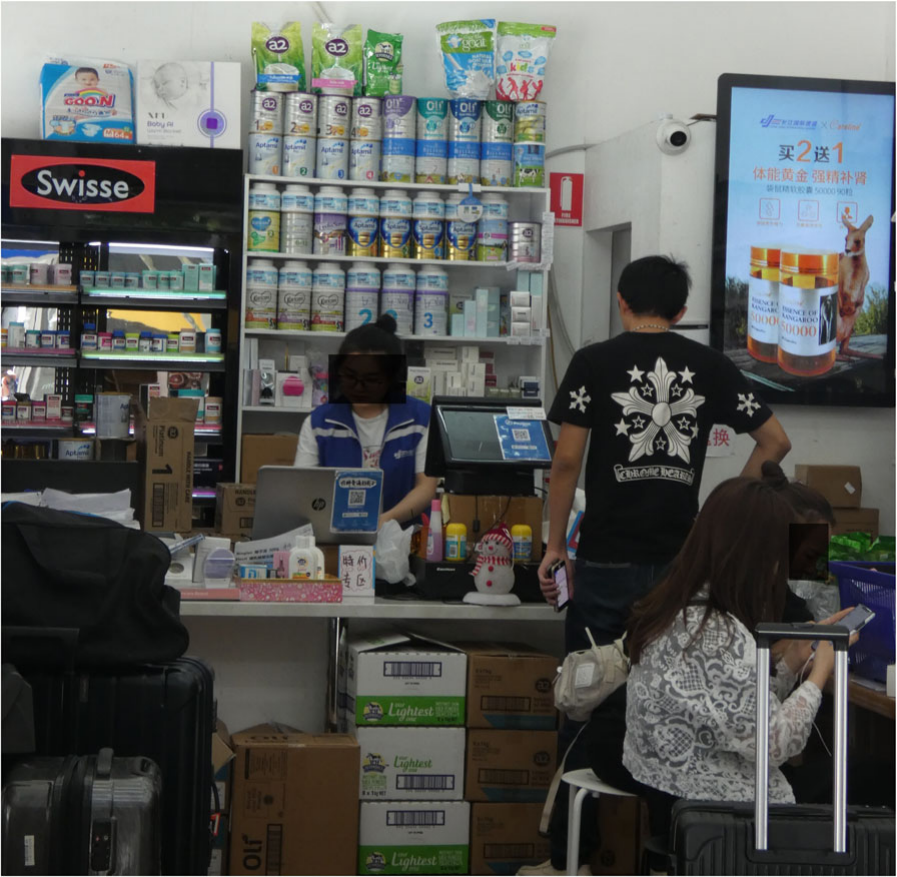
International distribution shop in Swanston St, Melbourne, Australia, October 2019

New Zealand is now the world’s largest exporter of dairy products, much of it the ‘white gold’ baby milk powder transported to China and South East Asian countries for sale. Australia is also a major pathway for the product to China and Asia, including via the active ‘daigou’ informal exporters who secure it by clearing supermarket shelves in Australia and New Zealand. These shops are set up to package and transport the products from Australia to China “(see Fig. 1)”.

The minimal regulatory constraints on the false and misleading marketing of these products and their health or nutrition claims [40–42] in Australia and New Zealand under an industry self-regulatory agreement (known as the MAIF Marketing Agreement on Infant Formula MAIF) is not applied at all to marketing in export markets [43]. So, for example, Chinese mothers are seen as fair game for aggressive Australasian marketing that would not be acceptable even under Australasia’s weak self-regulatory industry agreement, MAIF [36, 38].

Medical bribery in China has meanwhile, been shown to be rife [44–46]. Two US companies have been convicted and fined for such activities there [47]. Australasian governments by contrast simply rejoice in the sales and the boost to international trade [48]. In many countries there are no laws making exporters responsible for their illegal or unethical marketing activities overseas. Tobacco industry techniques are now known to be used by Big Formula as well as Big Tobacco [49].

With the latest data on GHG from these health and environmental experts on escalating sales in China and nearby countries, the chickens may begin to come home to roost. Climate change is increasingly evident as a reality, even a 1.5 °C global temperature rise will see several regional changes in climate, including extreme temperatures, floods or droughts, and increased climate-related risks to health, livelihoods, food security, water supply, human security, and economic growth [32].

This IYCF policy blindness and lethargy means opportunities for both mitigating and ameliorating climate change strategies are lost or neglected. Investing in breastfeeding will create populations that are less vulnerable to disruptions such as natural disasters, extreme weather events and humanitarian emergencies caused by climate change. Formula feeding is highly problematic in all country income settings, but especially when utilities such as water supply and electricity become suddenly unavailable.

## Conclusion

Global environmental change threatens food security and increases undernutrition: the very young feature among those most at risk. The Lancet Commission on Planetary Health [50] has identified that dietary shifts including to processed foods (which include milk formula [11]) are contributing both to disease burdens and greenhouse gas emissions. Promoting dietary change (for example away from animal products) could improve nutrition and health as well as reduce greenhouse gas emissions. Adaptation and building resilience is necessary to reduce vulnerabilities.

Breastfeeding protection, support and promotion helps to safeguard planetary and human health by minimising environmental harm, and is a beneficial response to concerns about diet related disease burdens and climate change. Formula feeding is a maladaptive practice in the face of contemporary global environmental and population health challenges.

The WHO UNICEF Global Strategy on Infant and Young Child Feeding sets out the now well-established effective policies and interventions for improving breastfeeding and optimal IYCF - such as paid maternity leave, BFHI and WHO International Code implementation [51, 52]. This tried and tested package of measures presents a rare opportunity to both reduce the greenhouse gas problem and improve human nutrition, health, and health equity. It also has strong economic foundations.

It is well past the time for urgent discussions about how reducing the unnecessary promotion, use and societal costs of formula milk feeding by investing in breastfeeding can help tackle the greatest challenge humanity has ever faced - sustaining Mother Earth. Economics and environment as well as human health and well-being, make investing in breastfeeding a priority in all country settings.

## Data Availability

Not applicable.
